# Effect and mechanism of the improvement of coastal silt soil by application of organic fertilizer and gravel combined with *Sesbania cannabina* cultivation

**DOI:** 10.3389/fpls.2022.1092089

**Published:** 2022-12-22

**Authors:** Xiaochi An, Menglin Sun, Kaiyan Ren, Min Xu, Zaifeng Wang, Ying Li, Hailong Liu, Bin Lian

**Affiliations:** College of Marine Science and Engineering, College of Life Sciences, Nanjing Normal University, Nanjing, China

**Keywords:** muddy coast, saline-alkali soil, co-occurrence network, bacterial diversity, sand, serpentine

## Abstract

Jiangsu Province of China has a large area of coastal silt soil (CSS) with poor permeability, high salinity, and poor nutrients, which brings great difficulties to the development and utilization of coastal zones, so that needs to be improved as a matter of urgency. In this study, river-sand, serpentine, and organic fertilizer were used as additives in CSS, and *Sesbania cannabina*, a salt-tolerant cash crop, was planted in these differently treated soils. Through high-throughput sequencing, analysis of soil physico-chemical properties, and detection of plant growth status, the rhizosphere bacterial diversity of *S. cannabina* growing in CSS under different treatments and their environmental impact factors were studied, while exploring the effect and mechanism of organic fertilizer combined with gravel as a CSS modifier. The results implied that single application of organic fertilizer could significantly increase the fertility levels of total nitrogen (TN), total organic carbon (TOC) and Avail. P in CSS; then, the application of organic fertilizer with river-sand significantly reduced salt content and alkalinity of soil; meanwhile, in the treatment of single application of organic fertilizer and application of organic fertilizer combined with river-sand, the rhizosphere of *S. cannabina* enriched the bacterial communities of organic matter degradation and utilization to varying degrees. The soil moisture content and indicators related to saline-alkali soil (including total salt, electrical conductivity (EC), exchangeable sodium percentage (ESP), Avail. Na and Avail. K, *etc*.) were further reduced significantly by the application of organic fertilizer combined with river-sand and serpentine. The method has greatly improved the growth conditions of *S. cannabina* and promoted the positive development of its rhizosphere bacterial community. Among them, in the treatment of organic fertilizer combined with river-sand and serpentine, a variety of plant growth-promoting rhizobacteria (PGPR, such as *Sphingomonas*, *Ensifer*, and *Rhodobacter*) and nitrogen-cycle-related bacteria (such as nitrate-reduction-related bacteria, nitrogen-fixing bacteria like *Ensifer*, and purple non-sulfur photosynthetic bacteria like *Rhodobacter*) were enriched in the rhizosphere of *S. cannabina*; moreover, the mutual association and robustness of bacterial co-occurrence networks have been significantly enhanced. The results provide a theoretical basis and reference model for the improvement of coastal saline-alkali silt soil.

## 1 Introduction

High salinity accelerates soil degradation, which is one of the main stressors affecting soil health, limiting plant growth and crop productivity (Rengasamy, 2010; Qin et al., 2016; Daliakopoulos et al., 2016; Wang et al., 2020). Due to high groundwater levels in coastal areas, a large number of soluble salts migrate to the surface soil and accumulate to form typical coastal saline-alkaline soils with the rising and evaporation of groundwater (He et al., 2020). The coastal areas of China have more than 20,000 km^2^ of coastal saline-alkali land (Cui et al., 2021), of which the muddy saline-alkali silt soil is the most difficult to use. Some 97% of the coastline in Jiangsu Province are muddy coasts. These coastal silt soils (CSSs) are subject to severe salinization, poor nutrition, and water permeability, so are difficult to improve (An et al., 2022). The research into the restoration techniques for CSS is of great significance to alleviate the shortage of land resources and the construction of coastal ecological landscapes.

A variety of soil amendments have been proved to be available for improvement of saline-alkali soil. For example, organic fertilizer can promote the formation and stability of soil aggregates in saline-alkali soil (Schmidt et al., 2011; Zhang et al., 2013), increase soil nutrient content and biological abundance, and reduce soil salt content (Wu et al., 2013; Zhang et al., 2022). In the practice of CSS improvement, it was found that the CSS with high sediment content in the same area had greater air permeability and lower salt content, which indicated that adding sand helped to improve the physico-chemical properties of CSS. However, with strict control of the local sand mining industry, the source of river-sand and sea-sand is a prominent problem. The reserves of serpentine deposits are extremely rich in Donghai County, Lianyungang City, Jiangsu Province (Lu et al., 2022). At present, a large amount of low-quality serpentine mining waste has not been effectively utilized. Considering that serpentine is rich in mineral elements such as magnesium (Galey et al., 2017; Mpouras et al., 2017; Sakaguchi et al., 2018), it may be effective when trying to improve local saline-alkali soil by using an appropriate amount of serpentine instead of sand. In addition, the weathering of serpentine coupled with carbon fixation is also expected to increase the capture of atmospheric CO_2_ (Kojima et al., 1997; Liu et al., 2021; Lu et al., 2022), which may increase more soil carbon sinks while improving saline-alkali land. However, the application of serpentine in CSS has not been reported, thus the application of organic fertilizer combined with sand and serpentine in CSS improvement is warranted to be scientifically tested.

Although the addition of nutrients and amendments such as organic fertilizer and sand can directly affect soil structure and properties, enhancement of soil nutrient accumulation through plant growth is also necessary (Wang et al., 2017; Zhao et al., 2020; Zhang et al., 2022). In fact, many salt-tolerant plants have the bio-remediation effect on saline-alkali soil. For example, *Sesbania cannabina*, *Medicago sativa*, *Sorghum sudanense*, *Festuca arundinacea*, and other salt-tolerant forage crops can increase the organic matter content of saline-alkali soil and contribute to soil desalination (Li et al., 2016; Bhattarai et al., 2020; Abdel-Rahman, 2017; Bañuelos and Beuselinck, 2003). The author planted a variety of salt-tolerant plants in the CSS in Lianyun New-Town, Lianyungang City, Jiangsu Province for two consecutive years, and found that *S. cannabina*, a leguminous plant, grows best in these areas. *Sesbania* is a high-quality forage crop with rich protein content, which can increase the soil nitrogen content (Anita and Sridhar, 2020). The famous Sesbania gum (a kind of biopolymer adhesive) can be extracted from *Sesbania* and can be used in food, textile, mining, energy, medicine, sewage treatment, and other industries (Shi et al., 2014; Pinki et al., 2018; Shi et al., 2019; Wang et al., 2022). Therefore, planting *S. cannabina* to participate in the restoration of saline-alkali land has the potential for application in practice.

Bacteria are major participants in soil biogeochemical processes (Fitter et al., 2005; Nielsen et al., 2011), which are characterized by rapid growth and strong variability, can quickly adapt to changes in the environment. Therefore, bacterial diversity is an important biological indicator reflecting environmental disturbance and changes in the quality of ecosystems (Bouchez et al., 2016; An et al., 2022).

In this study, *S. cannabina* was planted on CSS which was improved by adding organic fertilizer, river-sand, and serpentine. The rhizosphere bacterial diversity and soil physico-chemical properties of *S. cannabina* in different improvement treatments were studied, to explore the improvement effects and mechanism of the combined improvement model involving application of organic fertilizer, gravel and growth of *S. cannabina* on the CSS. This result provides a theoretical basis for the ecological utilization of muddy saline-alkali silt soil.

## 2 Materials and methods

### 2.1 Description and design of field test plot

The field test plot is sited in the coastal area of Lianyun New-Town, Lianyungang City, Jiangsu Province (E119°22′, N34°76′). The climate is of the warm temperate type, with a sub-tropical transitional humid monsoon climate (http://www.weather.com.cn/cityintro/101191001.shtml); the soil is of typical CSS type. The CSS in this plot is formed by hydraulically transported and filled by dredger and mud pump from the bottom of beach in coastal zone in the process of reclamation project (An et al., 2022), with characteristically high salinity, viscosity and moisture content.

From July to August 2020, the soil of the test plot was improved by adding exogenous substances and planting salt-tolerant plants. The implementation scheme of CSS improvement by adding different exogenous substances is displayed in **Table 1**, in which three repetitions are set for each sample plot (2 m * 4 m). During the implementation of the scheme, the first 200 mm depth of soil on the surface layer of the sample plot was first ploughed, then the exogenous additives were added before being ploughed again, evenly mixing the additives and soil. Among them, the organic fertilizer is provided by Lianyungang Golden Coast Development and Construction Co. Ltd, Lianyungang City, and the nutrient composition is found to be as follows: TOC 3.19%, total nitrogen (TN) 28.220 g·kg^-1^, alkali-hydro nitrogen 4.865 g·kg^-1^, available potassium 1.035 g·kg^-1^, available phosphorus 2.315 g·kg^-1^, moisture content 15.14%, pH 6.77; the sand is local river-sand of Lianyungang city, with the grain diameter ranging between 1 and 2.5 mm; mineral powder of serpentine was purchased from the serpentine mining area in Donghai County, Lianyungang City, with the grain diameter ranging between 1 and 2.5 mm. In addition, the salt-tolerant plants *S. cannabina, Medicago sativa, Limonium bicolor*, and *Amaranthus tricolor* were planted in the untreated CSS of the sample plot for two consecutive years previously, of which only *S. cannabina* could grow well and form a single vegetative community. Based on this, *S. cannabina* was used as an experimental species in CSS treated with different exogenous substances, and the planting density was about 240 plants·(m^2^)^-1^.

**Table 1:** Grouping information of CSS improved by adding different exogenous substances.

Group	Exogenous substance			Plant
	Organic fertilizer(6.75 kg·m^-3^)	River-sand(30 kg·m^-3^)	Serpentine(30 kg·m^-3^)	*Sesbania cannabina*(6g·m^-3^)
CK	–	–	–	+
OF	+	–	–	
FS	+	+	–	
SS	+	+	+	

+ indicates that this substance is added to this group; - denotes that this substance is not added to this group.

### 2.2 Sample collection

From October to November 2020, rhizosphere soil and soil around the roots of *S. cannabina* and the bare soil in four sample plots were collected, with three replicates for each group of samples. Meanwhile, for each repetition of the sample, the soil with the same weight of the five adjacent sampling points was selected and mixed evenly. Specific sampling methods include: vertically pulling out the plants, shaking off the bulk soil attached to the roots, and the soil attached to the surface of the root of the *S. cannabina* was collected in a sterile sampling bag with a sterilized brush, which is the rhizosphere soil of *S. cannabina*, for total DNA extraction and high-throughput sequencing; the sterilized shovel was adopted to collect the soil around the *S. cannabina* roots within a 100-mm radius centered on the root and the bare soil without plant growth, forming samples of the soil around the roots of *S. cannabina* and the bare soil respectively, for determination of soil physico-chemical properties; in addition, a 100 cm^3^ ring knife was utilized to collect the soil around the *S. cannabina* roots within 100 mm, for determination of porosity. The depth of all the collected soil samples ranged between 50 and 150 mm. The collected samples were stored and transported in dry ice, and frozen at -80 °C. At the same time, the whole plants of *S. cannabina* grown on the ground of four sample plots were collected, and each group of plant samples contained six replicates. The dry biomass of the plants was measured after drying (at 45°C).

### 2.3 Determination of soil physico-chemical properties

The soil around the roots were ground (to pass through a 60-mesh sieve) after air drying, and the detection methods of soil physico-chemical properties included: soil mineral crystal composition was determined by using an X-ray crystal diffractometer (Olympus, BTX-526, USA); total salt was measured by weighing deionized soil suspension with a soil to water ratio of 1:5 (w/v) (Lv and Li, 2010); electrical conductivity (EC) was identified by conductivity meter (SX731, Shanghai Sanxin, China) using deionized soil suspension with a soil water ratio of 1:2 (w/v); the cation exchange capacity was measured by referring to Chinese National Standard NY/T 1121.5-2006; exchangeable sodium (exchange. Na), potassium (exchange. K), magnesium (exchange. Mg), and calcium (exchange. Ca) were detected by referring to Chinese National Standard NY/T 1615-2008; exchangeable sodium percentage (ESP) denotes the percentage of exchangeable sodium content in cation exchange capacity, also known as alkalinity (Richards, 1954); TN was measured according to Chinese National Standard LY/T 1228-2015; total organic carbon (TOC) was measured according to Chinese National Standard HJ 695-2014; the detection of soil available potassium (Avail. K), available phosphorus (Avail. P), and available magnesium (Avail. Mg) were detected by referring to Chinese National Standard NY/T 889-2004/3.1, NY/T 1121.7-2014, and LY/T 2445-2015, respectively; other available metal ions were extracted by AB-DTPA method and determined by inductively coupled plasma atomic emission spectrometer (Prodigy Plus, Leeman, USA) (Soltanpour, 1985; Malathi et al., 2018). The soil around roots collected by ring-cutter was used for the following determinations: moisture content (MC), soil bulk density (SBD) in accordance with Chinese National Standard NY/T 1121.4-2006; the specific gravity was measured with reference to Chinese National Standard NY/T 1121.23-2010; porosity was calculated thus;


Soil porosity (%) = (1− SBD/specific gravity) × 100

### 2.4 Soil total DNA extraction and high-throughput sequencing

The total genomic DNA of organisms in soil samples were extracted by the CTAB method (Minas et al., 2011), and then the 16S rRNA gene V3-V4 hypervariable region in bacterial genomic DNA was amplified using V3-V4 universal primers. The sequence of V3-V4 universal primers were 341F-5’-CCTAYGGGRBGCASCAG-3’; 806R-5’-GGACTACNNGGGTATCTAAT-3’. DNA library construction was conducted using the DNA library construction kit (TruSeq^®^ DNA PCR-Free Sample Preparation Kit). After the library was qualified, high-throughput sequencing was conducted on the Illumina NovaSeq6000 platform of Beijing Novogene Technology Co., Ltd, China.

### 2.5 Data processing and statistical analysis

The physico-chemical properties of soil samples were statistically analyzed in SPSS 22.0 (IBM, US) software and further descriptive, variance, and Spearman correlation analysis were performed. The data between multiple groups were statistically tested by Duncan’s significance test (*P<* 0.05). Linear fitting of the data was undertaken using Origin 9.0 software (OriginLab, USA).

To achieve the readability of high-throughput sequencing data, the sequencing data processing was presented as follows: according to the Barcode sequence and the PCR amplification primer sequence, the sample data were separated from the off-line data; with the barcode and primer sequences removed, Flash V1.2.7 software (Magoč and Salzberg, 2011) was used to splice the reads sequences of each sample, and the spliced sequence then contained the raw rags; these were strictly filtered using Qiime V1.9.1 (Bokulich et al., 2013) to obtain high-quality tags (clean tags); clean tags removed chimeric sequences using Vsearch 1.2.5 software (Haas et al., 2011; Rognes et al., 2016), to obtain the final effective tags. Operational taxonomic unit (OTU) clustering and species annotation of effective tags: Uparse V.0.1001 software was used to cluster effective tags with 97% identity into OTUs (Edgar, 2013); species annotation analysis of OTU sequences was performed using the SSUrRNA database (http://www.arb-silva.de/) (Wang et al., 2007).

The visualization analysis of high-throughput sequencing data was described as follows: the indices of bacterial alpha diversity (*e.g.* Observed-species, Chao1, Shannon, Simpson) were calculated by using Qiime software; FAPROTAX software was used to predict bacterial function. Various packages in R 3.2.3 software were used for different analyses as follows: Venn diagrams were plotted using the Venn package; principal component analysis (PCA) diagrams and histograms of bacterial community composition were plotted using the ggplot package; significance testing of difference (*T*-test) among bacterial communities of different samples was analyzed using the rstatix, reshape2, and dplyr packages; redundancy analysis was undertaken using the vegan package; heatmap analysis was performed using the Hmisc and pheatmap packages. The WGCNA package in software R and Gephi 0.9.2 software were jointly used to study the bacterial co-occurrence network, in which the points in the network are bacteria at the genus level, and the network did not contain genera with the number of sequenced sequences less than, or equal to, 10 in different replicates; robustness calculation of the co-occurrence network was performed by R 3.2.3 software (Yuan et al., 2021). SPSS 22.0 software and SPSS Amos 24.0 software (IBM, US) were jointly employed to establish the requisite structural equation model (SEM).

## 3 Results

### 3.1 Physico-chemical properties of soil and growth characteristics of *S. cannabina* in CSS with different treatments

**Figure 1:**
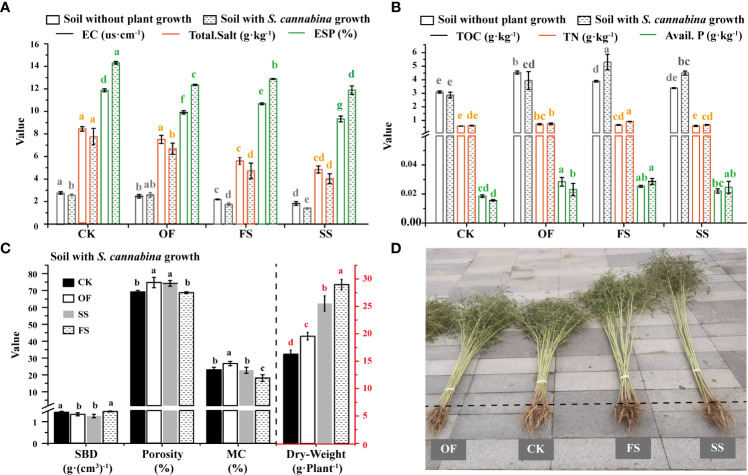
**(A)** Saline-alkali-related chemical properties in CSS with different treatments; **(B)** Nutrient-related chemical properties in CSS with different treatments; **(C)** Physical characteristics of soil around *S. cannabina* root and biomass of *S. cannabina* grown in CSS with different treatments; **(D)** Photos of *S. cannabina* grown in CSS with different treatments. (The different lowercase letters of the same index in different sample groups indicate that there is a significant difference between the groups in this index [according to Duncan’s significance test, *P<* 0.05)].

The effects of different soil exogenous treatments on soil environment were significant, and indirectly affected the growth of *S. cannabina*. The analysis of soil mineral composition after different improvement treatments (**Figure S1**) showed that the addition of organic fertilizer and river-sand did not change the main mineral composition of the soil, which was still dominated by quartz, muscovite, and albite; the additional serpentine is chrysotile, and the chemical formula is Mg_3_[Si_2-_ *_x_* O_5_](OH)_4-4_ *_x_*. Firstly, the chemical characteristics of soil in different treatment sample plots were investigated as shown in **Figures 1A** **, B**. No matter whether *S. cannabina* grows, the order of soil saline-related environmental factors (total salt, EC) content in different treatment sample plots is such that CK > OF > FS > SS, and the order of soil alkalinity in different treatment sample plots is CK > FS > OF > SS (**Figure 1A**). In the bare soil without *S. cannabina* growth, the order of fertility contents in different treatment sample plots is OF > FS ≈ SS > CK; however, affected by the growth of *S. cannabina*, the order of fertility contents in different treatment sample plots is FS > SS ≈ OF > CK (**Figure 1B**). Secondly, the physical properties of the soil around the *S. cannabina* root (**Figure 1C**) are as follows: compared with the sample of CK, the sample OF had significantly increased soil porosity and moisture content (MC), but the soil bulk density (SBD) decreased; compared with OF, the sample of FS had significantly reduced soil MC; compared with the group of FS, the sample of SS had significantly decreased soil porosity and MC, and increased SBD. In addition, the order of biomass of *S. cannabina* growing in different treatment sample plots is such that SS > FS > OF > CK (**Figures 1C, D**).

Coastal areas are affected by saline intrusion, of which saline-alkali is the main stressor affecting agricultural production and ecology of coastal soil. Therefore, the specific components of salt-alkali-related cations in the soil around *S. cannabina* root under different improvement treatments were detected (**Table 2**). The results showed that the contents of available cations and exchangeable cations in SS were significantly different from those in other treatments as follows: the contents of available base ions (Na, K) and trace elements (Fe, Mn, Cu) decreased significantly, while the contents of available Mg, Ca, and Ni increased significantly; the total amount of cation exchange in SS was significantly reduced, accompanied by significant decreases of exchangeable Na, K, and Ca, while the content of exchangeable Mg was remarkably increased.

**Table 2:** Contents of base ions and trace elements in the soil surrounding *S. cannabina* grown in CSS with different treatments.

Index	CK	OF	FS	SS
Available elements (g·kg^-1^)	/	/	/	/
Avail. Na	1.172 ± 0.003 a	1.172 ± 0.001 a	1.163 ± 0.011 a	1.084 ± 0.035 b
Avail. K	1.007 ± 0.095 a	1.013 ± 0.086 a	0.858 ± 0.077 b	0.514 ± 0.135 c
Avail. Ca	0.223 ± 0.010 b	0.246 ± 0.021 b	0.240 ± 0.019 b	0.294 ± 0.023 a
Avail. Mg	0.700 ± 0.041 b	0.677 ± 0.038 bc	0.637 ± 0.016 c	0.883 ± 0.023 a
Avail. Fe	0.221 ± 0.021 ab	0.229 ± 0.015 ab	0.257 ± 0.021 a	0.191 ± 0.027 b
Avail. Mn	0.0251 ± 0.004 a	0.024 ± 0.003 a	0.028 ± 0.004 a	0.011 ± 0.002 b
Avail. Ni	0.0005 ± 0.0001 b	0.0006 ± 0.0000 b	0.0009 ± 0.0001 b	0.0233 ± 0.0014 a
Avail. Cu	0.006 ± 0.000 a	0.006 ± 0.001 a	0.007 ± 0.000 a	0.004 ± 0.001 b
Exchangeable elements (cmol·kg^-1^)	/	/	/	/
Exchange. Na	2.405 ± 0.027 a	2.040 ± 0.017 b	2.073 ± 0.006 b	1.807 ± 0.059 c
Exchange. K	0.813 ± 0.031 a	0.799 ± 0.024 a	0.717 ± 0.012 b	0.510 ± 0.046 c
Exchange. Ca	9.635 ± 0.031 b	11.373 ± 0.491 a	9.767 ± 0.133 b	8.812 ± 0.074 c
Exchange. Mg	2.054 ± 0.011 c	2.054 ± 0.024 c	2.301 ± 0.030 b	3.150 ± 0.060 a
Cation exchange capacity	16.801 ± 0.057 a	16.512 ± 0.067 b	16.097 ± 0.085 c	15.197 ± 0.115 d

The different lowercase letters of the same index in different sample groups indicate that there is a significant difference between the groups in this index (according to Duncan’s significance test, *P*< 0.05).

### 3.2 Variations in bacterial community structure in the rhizosphere of *S. cannabina* under different treatments

#### 3.2.1 Analysis of bacterial diversity

**Figure 2:**
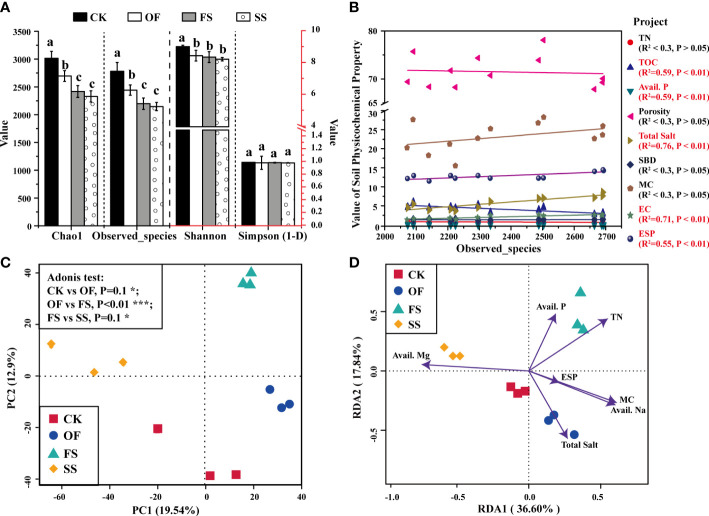
**(A)** Bacterial alpha diversity indices of *S. cannabina* rhizosphere in CSS with different treatments (the different lowercase letters of the same index in different sample groups indicate that there is a significant difference between the groups in this index [according to Duncan’s significance test, *P<* 0.05)]; **(B)** Linear fitting relationship between environmental factors and bacterial alpha diversity; **(C)** Principal component analysis diagram shows the difference of bacterial community among different samples; **(D)** Redundancy analysis diagram shows the correlation analysis between bacterial communities and environmental factors in different samples.

Alpha diversity indicates the complexity of the sample, which can assess the bacterial richness and evenness of each sample habitat (**Figure 2**). According to the Chao1, Observed_species, and Shannon indices showed that the bacterial richness and evenness of OF, FS, and SS were significantly lower than CK; in addition, the bacterial richness of FS was similar to that of SS, which was significantly lower than that of OF (**Figure 2A**). Furthermore, the linear fitting of soil environmental factors and observed_species was performed as shown in **Figure 2B**. The result showed that the soil saline-related environmental factors (total salt, EC) had the highest linear fitting degree with the bacterial (*R* ^2^ > 0.7), and there was a very significant positive correlation between them (*P*< 0.01); there was a very significant positive correlation between alkalinity-related environmental factor (ESP) and bacterial richness (*R* ^2^ > 0.5, *P*< 0.01); however, the soil nutrient content (TOC, Avail. P) was significantly negatively related to the bacterial richness (*R* ^2^ > 0.5, *P*< 0.01).

The samples with similar or different bacterial abundances are composed of different bacterial community structures, as shown in **Figures 2C**, **S2**. The Venn diagram (**Figure S2**) showed that there were only 1818 common OTUs among the four groups of 5107 OTUs. Meanwhile, principal component analysis (PCA) could reflect the diversity of bacteria among different treatment groups, as shown in **Figure 2C**. The results implied that the samples of OF, FS, and SS were divided into three relatively independent regions in the dimensions of PC1 and PC2, which indicated that there were differences in community composition among different sample groups; and then, the results of Adonis difference significance test showed that there were differences in community composition between CK *v*. OF and FS *v*. SS, while there were significant differences in bacterial community composition between OF *v*. FS (**Figure 2C**).

A redundancy analysis (RDA) model was used to determine the correlation between bacterial community composition and environmental factors (Li et al., 2015). In the RDA model, the included angle between the environmental factor represented by the arrow and the corresponding sample group represents the correlation between them (acute angle: positive correlation; obtuse angle: negative correlation; right angle: no correlation); at the same time, the greater the distance between the arrow and the origin, the stronger the correlation between the environmental factor and the corresponding sample. Based on this, the results (**Figure 2D**) showed that the bacterial community composition of OF was significantly positively correlated with total salt (*P*< 0.01); the bacterial community composition of FS was significantly positively correlated with TN (*P*< 0.01); the bacterial community composition of SS was positively correlated with Avail. Mg (*P*< 0.1), and negatively correlated with Avail. Na and MC (*P*< 0.1).

#### 3.2.2 Analysis of bacterial community composition and biomarker

**Figure 3:**
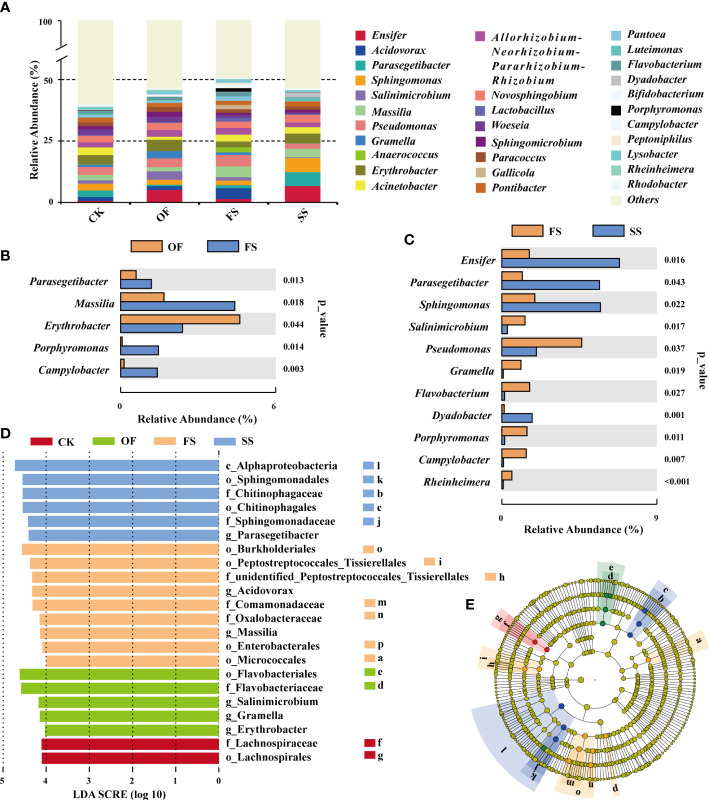
**(A)** Histogram of TOP-30 dominant bacterial community composition in genus level at different samples; **(B, C)** Analysis of the differences (*T*-test) in the abundance of TOP-30 dominant genera in different samples (CK *v*. OF no significant difference genus; **(B)** OF *v*. FS; **(C)** FS *v*. SS); **(D, E)** LEfSe analysis indicates bacteria with dominant abundance in different samples at different taxonomic levels, **(D)** The distributed histogram of LDA value shows the bacteria with LDA score > 4, which namely biomarker (the prefixes o_, c_, f_, and g_ of bacterial name represent the order, class, family, and genus of different taxonomic levels of bacteria respectively); **(E)** Biomarker evolutionary cladistic diagram with statistically significant differences between different samples (the circle radiating from inside to outside represents the taxonomic level from phylum to family; biomarker is colored with the different samples; different lowercase letters in the figure represent different bacteria, where the names of these bacteria are shown in **Figure 3D**).

Bacterial abundance will determine the functional role in complex bacterial communities (Rivett and Bell, 2018). The ecological functions of dominant bacterial groups can reflect the interaction between host, soil microenvironment, and bacteria to some extent. The sequences were classified from phylum to species, and the composition of dominant bacterial communities of TOP-30 is shown in **Figure 3A**. Among them, the dominant genera (abundance > 0.8%) in the four groups of samples included *Sphingomonas*. *Massilia*, *Pseudomonas*, *Erythrobacter*, *Acinetobacter*, *Allorhizobium - Neorhizobium - Pararhizobium - Rhizobium*, *Novosphingobium*, *Paracoccus*, *Pontibacter*, and *Sphingomicrobium*. The results shown in **Figures 3B, C** showed the dominant bacterial genera with significant differences in abundance between each sample group, among which there was no significant difference between the sample of CK and OF. There were significant differences between the samples of FS and OF: *Parasegetibacter*, *Porphyromonas*, and *Campylobacter* were dominant in FS; *Massilia* and *Erythrobacter* were dominant in FS and OF, but the abundance of *Massilia* in FS was significantly increased, while the abundance of *Erythrobacter* was significantly decreased (**Figures 3A, B**). Compared with FS, the sample of SS showed a significantly increased abundance of *Dyadobacter* and *Rhodobacter*; *Salinimicrobium*, *Gramella*, *Flavobacterium*, *Porphyromonas*, and *Campylobacter*, as the dominant genera of FS, decreased significantly in SS; the genera of *Ensifer*, *Parasegetacter*, *Sphingomonas*, and *Pseudomonas* were dominant in FS and SS, but the abundance of *Ensifer*, *Parasegetibacter*, and *Sphingomonas* increased remarkably in SS, while the abundance of *Pseudomonas* decreased significantly (**Figures 3A, C**). These results implied that the soil improvement treatment with gravel had a significant impact on the composition of the dominant bacteria community in the rhizosphere of *S. cannabina*.

Linear discriminant analysis effect size (LEfSe) is an analytical tool used to discover and interpret high-dimensional biomarkers. By comparing multiple samples, it is possible to find statistically different biomarkers between different samples (Segata et al., 2011), which will be an important basis for explaining the functional changes of bacterial communities in different samples. The results of LEfSe (**Figures 3D** **, E**) showed that the sample of FS had the largest number of biomarkers, and the biomarkers showing FS at the minimum evolutionary taxonomic level were o_Micrococcales, f_unidentified_Peptostreptococcales_Tissierellales, g_*Acidovorax*, g_*Massilia*, o_Enterobacterales; biomarkers of the sample of SS were f_Chitinophagaceae, f_Sphingomonadaceae, g_*parasegetibacter*; of the sample of OF included g_*Salinimicrobium*, g_*Gramella*, g_*Erythrobacter*; the biomarker of the sample of CK was f_Lachnospiraceae.

### 3.3 Variation of bacterial function and network structure in the rhizosphere of *S. cannabina* under different treatments

#### 3.3.1 Predicted functions of bacterial communities

**Figure 4:**
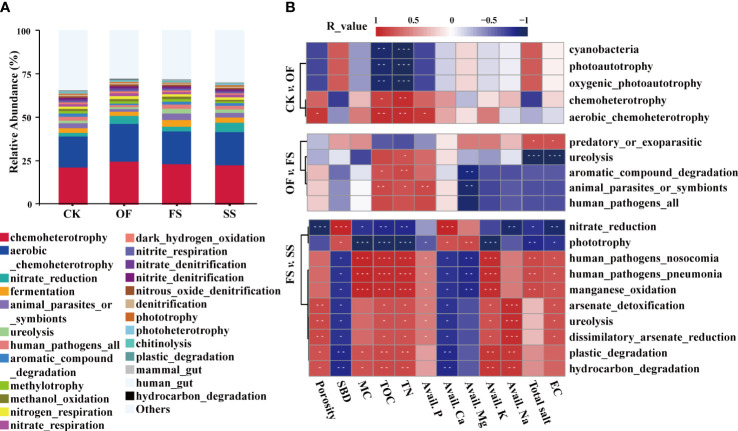
**(A)** Histogram of relative abundance of TOP-20 predicted function of bacterial community in different samples based on FAPROTAX; **(B)** Spearman correlation analysis between bacterial predicted function and environmental factors with significant differences among different samples (“*” denotes a *P*-value< 0.1, “**” denotes a *P*-value< 0.05, “***” denotes a *P*-value< 0.01).

Functional diversity is an important link between biodiversity and ecosystem functions (Escalas et al., 2019), and different bacterial communities explain different ecosystem functions (Wagg et al., 2019). The functional annotation of prokaryotic taxa (FAPROTAX) exerts a good predictive effect on the biochemical cycle process of environmental samples (Louca et al., 2016). Based on the FAPROTAX database, the function of bacterial communities in different samples was predicted, and the results of TOP-20 predicted function are shown in **Figure 4A**. The bacterial communities in different samples have similar biochemical cycle functions. Among them, chemoheterotrophy and aerobic_chemoheterotrophy were the dominant functions; and then, many N and C metabolism-related functions have been annotated. The N metabolism-related functions included ureolysis and denitrification-related functions (nitrate_reduction, nitrogen_respiration, nitrate_respiration, nitrate_densitization, nitrate_densitization, nitrous_oxide_denitrification, and denitrification), and their relative abundance was more than 0.8%; the C metabolism-related functions included aromatic_compound_degradation, methylotrophy, methanol_oxidation, dark_hydrogen_oxidation, fermentation, phototrophy, photoheterotrophy, etc., their relative abundance exceeded 0.5%. These results showed that the C/N metabolism of the bacterial communities in the rhizosphere of *S. cannabina* was active with different exogenous treatments.

A variance analysis was performed on the dominant predicted function of bacterial communities between different samples (**Table S1**), and the effects of environmental factors on the different predicted functions were investigated (**Figure 4B**). Compared with the sample of CK, the function of chemoheterotrophy (including oaerobic_chemoheterotrophy) in the sample of OF was significantly enhanced, while the function of photoautotrophy (including cyanobacteria and oxygenic_photoautotrophy) was remarkably weakened. Among them, chemoheterotrophy was positively correlated with TOC and TN, and aerobic chemoautotrophy was also positively related to porosity and Avail. P; on the contrary, photoautotrophic was negatively correlated with TOC and TN. Compared with OF, the sample of FS had significantly enhanced functions of animal_parasites_or_symbionts, ureolysis, and aromatic_compound_degradation. Among them, ureolysis was positively correlated with TN, total salt, and EC; animal_parasites_or_symbionts and aromatic_compound_degradation were positively related to TOC and TN, while being significantly negatively correlated with Avail. Mg. Compared with FS, the sample of SS had enhanced the functions of nitrate reduction and phototrophy. Among them, nitrate reduction was significantly positively correlated with SBD and Avail. Ca, and significantly negatively correlated with TN, TOC, Avail. Na, EC, and porosity; phototrophy was positively correlated with Avail. Mg.

#### 3.3.2 Analysis of the topological characteristics of bacterial co-occurrence networks

**Figure 5:**
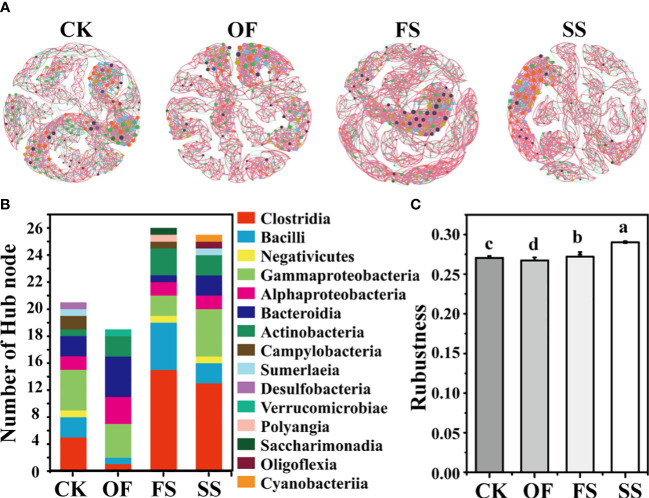
**(A)** Co-occurrence networks between bacterial taxa based on genus at the Class level according to the Pearson correlation coefficients (Nodes labeled with the same color indicate that different genera belonged to the same bacterial Class. Node size denotes link numbers with other nodes and larger sizes indicate more link numbers. The edge represents the connected line between two nodes with significant correlation (Pearson’s correlation coefficient > 0.6, *P*< 0.05). The red and green lines within the two nodes in the networks refer to positive and negative correlations, respectively). **(B)** The columnar stack diagram shows the distribution of the hub nodes of the bacterial co-occurrence network at the taxonomic level of class. **(C)** Robustness of the bacterial co-occurrence network in different samples (the different lowercase letters of the same index in different sample groups indicate that there is a significant difference between the groups in this index (according to Duncan's significance test, P < 0.05)).

The application of network theory to the study of bacterial communities can simulate the co-occurrence of bacteria. Bacterial co-occurrence networks can be adopted to discover bacterial relationships that are critical to the assembly or stability of the community and infer the effects of various interactions between bacterial communities on soil health and plant host development (Layeghifard et al., 2017). In this study, the bacterial co-occurrence network diagram (**Figure 5A**) of different samples had been established at the bacterial taxonomic level of genus, and the bacterial network structure of each sample was significantly different. Among them, the values of nodes, edges, average degree, and modularity of the bacterial co-occurrence network in the sample of CK were the highest, implying that the bacterial co-occurrence network in rhizosphere of *S. cannabina* grown in the original CSS was the most complex and tended to differentiate into different network modules (**Table 3**); however, the edge-based proportion of positive correlation in the bacterial co-occurrence network increased after adding different exogenous substances, and the order was FS > SS > OF > CK. This implied that different exogenous substances could promote the establishment of mutual association in the bacterial co-occurrence network (**Table 3**).

**Table 3:** Co-occurrence network properties within bacterial taxa in different samples in **Figure 5**.

Sample	Number of Nodes	Number of Edges ^a^	Proportion of Positive Edges	Average Degree ^b^	Average Path length ^c^	Average Clustering Coefficient ^d^	Modularity ^e^
CK	320	2952	58.435%	18.45	12.207	0.778	6.604
OF	293	2441	61.327%	16.662	12.319	0.777	3.914
FS	298	2609	65.315%	17.51	12.083	0.779	2.454
SS	271	2163	64.170%	15.963	12.113	0.793	2.696

a Edges can indicate the connection between two nodes.

b Degree denotes the number of nodes connected by a node to other nodes.

c Path length stands for the length of the shortest path between two nodes in the network.

d Clustering coefficients indicates the degree to which nodes tend to gather together.

e Modularity indicates the degree to which nodes tend to differentiate into different network modules.

The bacterial co-occurrence network can not only elucidate the global difference in bacterial community structure between different samples, but also show the importance of single bacteria in the whole community. Some bacteria as hub nodes (indicating nodes with high degree (> 25) and close centrality (> 0.10) in the network) may have an important ecological function in the bacterial community structure. The results (**Figure 5B**) showed that the number of hub nodes in different samples was ordered thus: FS ≈ SS > CK > OF, and the bacteria (class-level) to which the hub nodes belong were different (**Figure 5B**; **Tables S2–S5**). Compared with CK, the number of hub nodes belonging to Firmicutes (Clostridia, Bacilli, and Negativicutes) in the sample of OF decreased, while Bacteroidia and Actinobacteria increased. Compared with OF, the number of hub nodes belonging to Clostridia, Bacilli in the sample of FS increased, while the numbers of Alphaproteobacteria, Gammaproteobacteria, and Bacteroidia decreased. Compared with FS, the number of hub nodes belonging to Gammaproteobateria and Bacteroidia in the sample of SS increased, while Bacilli decreased. Different exogenous treatments significantly affected the hub bacteria of the bacterial co-occurrence network.

To determine whether and how different exogenous additives affect the stability of the bacterial co-occurrence network, a robustness analysis was performed to evaluate the stability of networks and their embedded members (Yuan et al., 2021; Sun et al., 2022). Robustness refers to the stability of the structure and function of a biological system when it is disturbed by uncertain factors. The greater the robustness, the stronger the system’s resistance to external disturbances (Yuan et al., 2021). The results (**Figure 5C**) showed that the robustness of the bacterial co-occurrence network in different samples was ordered thus SS > FS > CK > OF, indicating that organic fertilizer combined with gravel could promote the stability of the bacterial co-occurrence network in rhizosphere of *S. cannabina*.

## 4 Discussion

### 4.1 Effect and mechanism of organic fertilizer on improvement of CSS

Coastal saline-alkali silt soils are generally subjected to poor permeability and saline-alkali stress (An et al., 2022). According to the second national soil survey and relevant standards (National Soil Survey Office, 1998), the original soil on the experimental plot was subjected to severe salt stress (total salt > 6 g·kg^-1^), while the soil nutrients are at the sixth level of extreme deficiency (TN< 0.5 g·kg^-1^, TOC< 6 g·kg^-1^) (**Figure 1**). Therefore, with single application of organic fertilizer in CSS, the soil fertility and the biomass of *S. cannabina* were significantly improved (**Figures 1B** **–D**), improving the CSS.

Many studies (Fliessbach et al., 2007; Wang et al., 2016; Li et al., 2020) have shown that, short-term or long-term application of organic fertilizer in soil will lead to the improvement of soil microbial diversity. However, in this study, the Sesbania rhizosphere bacterial community of *S. cannabina* in the original CSS had a greater abundance (**Figure 2A**), more complex co-occurrence networks, and higher modularity (**Figure 5A**; **Table 3**). These results indicate that the single application of organic fertilizer causes sudden changes in soil physico-chemical characteristics (**Figure 2**), which may strengthen the adaptive screening based on specific functional bacterial species in the original soil microbial library. The transformation of bacterial community structure in the rhizosphere of *S. cannabina* was affected by the single application of organic fertilizer with the type of nutrient utilization as the screening model. Among them, some widely reported organic matter degrading bacteria were significantly enriched (**Figures 3D, E**), such as *Salinimicrobium* and *Gramella* (Yun et al., 2018; Voisin et al., 2020); meanwhile, Bacteroidia and Actinobacteria (Gurushankara et al., 2019; Voisin et al., 2020) became the hub node of the bacterial co-occurrence network (**Figures 5B**; **Tables S2–S5**), and the mutual association in the bacterial co-occurrence network increased; in addition, the functional proportion of chemoheterotrophic and phototrophic in rhizosphere bacterial community of *S. cannabina* was significantly increased (**Figure 4**; **Table S1**). The decomposition and utilization of soil organic matter forms the basis for soil bacteria to exert more biogeochemical cycling functions (Condron et al., 2010). In this study, the treatment with single application of organic fertilizer made more bacteria of organic matter decomposition and utilization screened and enriched, while these bacteria also played an important role in connecting the center of the bacterial network, which is conducive to soil organic matter activation and material circulation.

With single addition of organic fertilizer in CSS, the treatment can improve the soil fertility and significantly enrich functional bacteria that are conducive to the activation of soil organic matter; however, the moisture content has significantly increased (**Figure 1C**), and the soil salt stress has not been effectively mitigated (**Figure 1A**), which still needs further improvement.

### 4.2 Effect and mechanism of organic fertilizer combined with river-sand on improvement of CSS

Compared with the single application of organic fertilizer, in the treatment of organic fertilizer combined with river-sand (the simple case of FS), the moisture content and saline-alkali stress of soil were significantly decreased (**Figures 1A** **, C**); the growth of *S. cannabina* was further improved (**Figures 1C, D**). Meanwhile, functions of organic matter activation in the rhizosphere bacterial community of *S. cannabina* in FS were further enhanced (**Figure 4**; **Table S1**), including: functions of ureolysis and aromatic_compound_degradation of the bacterial community were significantly enhanced (**Table S1**); and then, the dominant bacteria of organic matter degradation had changed, such as *Parasegetacter*, *Porphyromonas*, o_Micrococcales, and f_unidentified_ Peptostreptococcales_ Tissirellales (Yan et al., 2020) and other bacteria were significantly enriched (**Figures 3A, B** **, D, E**). However, the treatment of FS increased functions of human pathogens and animal parasites _or_ symbionts, while the dominant bacteria such as *Campylobacter*, *Pantoea*, and the biomarker *Massilia* were reported as human pathogens (De Baere et al., 2004; Park and Shin, 2013). Hutchison et al. (2004; 2005) found that the application of organic fertilizer is one of the routes to contamination of soil by human pathogenic bacteria. In this study, the soil environment under the improved model of organic fertilizer combined river-sand may provide more suitable living conditions for these bacteria, which should draw attention to the relevant biological safety. Therefore, the quality management of organic fertilization regimes should be strengthened to prevent the introduction of pathogenic bacteria in large quantities with the application of organic fertilizer.

### 4.3 Effect and mechanism of organic fertilizer combined with river-sand and serpentine on improvement of CSS

**Figure 6:**
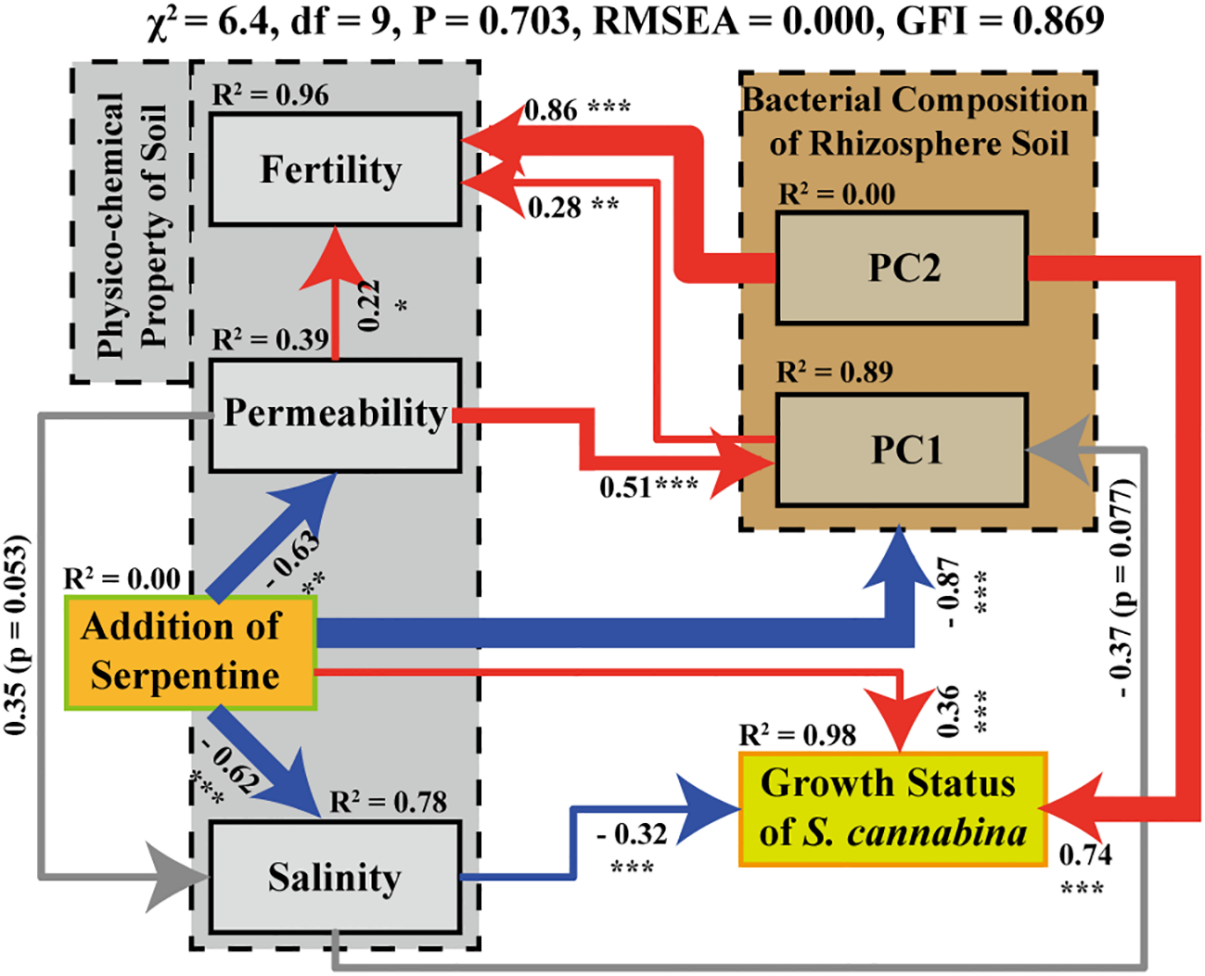
SEM model between the treatment of organic fertilizer combined with river-sand and serpentine and biotic or abiotic environmental variables. Among them, content of Avail. Mg reflects the treatment of SS (mainly as the addition of serpentine); Avail. Na, TN, and moisture content were used to reflect the physico-chemical indices of salinity, fertility, and permeability in soil respectively; dry-biomass reflect the growth status of *S. cannabina*; the fitted values of PC1 (distinguishing FS and SS) and PC2 (distinguishing OF and FS) in PCA analysis (**Figure 2C**) indicated the difference of bacterial community composition in different samples (the number marked on the connecting line between factors indicates the correlation between the two factors, and the larger the *R* ^2^, the stronger the correlation; the line thickness of the connecting line also reflects the magnitude of *R* ^2^; “***” on the line represents significance at *P* < 0.001, “**” represents significance at *P* < 0.01, and “*” represents significance at *P* < 0.05; the red and blue colors on a line represent positive correlation and negative correlation respectively).

The changes of soil properties such as soil salinity, fertility, and physical properties are mainly displayed among samples of different treatments (**Figures 1** **A–C**; **Table 2**). The application of organic fertilizer combined with river-sand and serpentine, as the sample of SS, highlights the crystal peak of serpentine in soil mineral crystals (**Figure S1**). According to the results of the mineral chemical composition analysis (based on X-ray fluorescence) of serpentine in the serpentine mining area of Donghai County, Jiangsu Province (Lu et al., 2022), the main components are MgO (37.84%), SiO_2_ (41.71%), Fe_2_O_3_ (14.37%), and CaO (3.29%). The weathering of minerals in SS may be the main reason for the significant increase of Avail. Mg and Exchange Mg in soil (**Table 2**). In addition, compared with FS, the moisture content and saline-alkali stress (Aval. Na, Aval. K, and Exchange Ca) of soil in SS were significantly reduced (**Figures 1A, B**, **Table 2**), and the biomass of *S. cannabina* was further significantly increased (**Figures 1C** **, D**). Simple linear model of RDA (**Figure 2D**) showed that the bacterial community composition in SS was correlated with Avail. Mg, moisture content, and Avail. Na. The covariance matrix of variables was used to study the correlation and multiple dependencies between multivariable (Lee, 2007; Larsson et al., 2020). Based on this, this study further estimated the causal relationship and multivariate correlation among the changes of environmental factors, the growth status of *S. cannabina*, and the rhizosphere bacterial community of *S. cannabina*, which are subjected to the influence of SS treatment, thus a structural equation model (SEM) between different factors was constructed, as shown in **Figure 6**.

The fitting data of SEM shown in **Figure 6** are good, as *χ* ^2^ ≥ 0, df ≥ 2, 0.05< *P*< 0.90, and RMSEA< 0.05, indicating that the model is reliable. SEM demonstrated that the application of organic fertilizer combined with river-sand and serpentine significantly improved permeability and salinity of soil and was directly and strongly correlated with the construction of rhizosphere bacterial community of *S. cannabina*. At the same time, the biomass of *S. cannabina* was promoted through direct and indirect effects associated with improving the salinity. Among them, the rhizosphere bacterial community of SS was significantly different from that of FS, which could be explained in the following four ways:

(1) Enriching weathered bacteria of serpentine with promoted plant growth. Organic acids secreted by root and some mineral-weathered microorganisms can promote mineral weathering (Archana et al., 2012; Lu et al., 2022). In this study, f_Sphingomonadaceae (*Sphingomonas*) is a biomarker in rhizosphere bacterial community of *S. cannabina* in SS (**Figures 3C** **, D**). Lu et al. (2022) found that *Sphingomonas* is usually enriched in the environment where serpentine is present and may participate in the weathering of serpentine. In addition, *Sphingomonas* is a widely reported PGPR growth under stress (*e.g.* drought, saline-alkali, and heavy metals) (Asaf et al., 2020).
(2) Enriching bacteria of the N-cycle with promoted plant growth. Weathering of serpentine increases the content of available Mg and Ni in SS (**Table 2**). This enriched Mg-content may be conducive to the enrichment of nitrogen-fixing bacteria in SS. The nitrogenase responsible for N2 fixation in nitrogen-fixing bacteria requires at least 16 molecules of Mg-ATP (conversion of one molecule of N2 to two molecules of NH3); the reaction equation (Seefeldt et al., 2009) is as follows:

$$N_{2}+\;8\;H^{+}+\;16\;Mg-ATP\;+\;8\;e^{-}\to \;2\;NH_{3}+\;H_{2}+\;16\;Mg-ADP\;+\;16\;Pi$$

In addition, Ni, at a low concentration, is the main component of [NiFe]-hydrogenase, which is used to recover H_2_, a by-product of N_2_ reduction in the nitrogen fixation process of legume rhizobia (Evans et al., 1988); meanwhile, Ni can mediate the up-regulation of ammonia transporter, the main metabolite of nitrogen metabolism (Liu et al., 2021), thereby promoting nitrogen cycle. In this study, the function of nitrate reduction in SS was significantly enhanced (**Figure 4**, **Table S1**), and the abundance of the rhizobium member *Ensifer* (Gopalakrishnan et al., 2015; Yan et al., 2016) that could attach to the root surface was significantly increased (**Figures 3A** **, C**). *Ensifer* has also been shown to increase photosynthesis and growth activity of plants (Peng et al., 2002; Chi et al., 2005; Toyama et al., 2021).

(3) Enriching photosynthetic bacteria with functions of promoting plant growth and nitrogen fixation using auxiliary. *S. cannabina*, a leguminous plant, has high nitrogen demand (Herridge et al., 2008). Except for nitrogen-fixing bacteria, most photosynthetic bacteria have nitrogen-fixing capacity, and their nitrogen-fixing capacity increases rapidly when they coexist with nitrogen-fixing bacteria (Ludden and Roberts, 2002). In this study, the phototrophic function in rhizosphere bacterial community of *S. cannabina* was significantly strengthened in SS (**Table S1**), which was significantly positively correlated with the Avail. Mg content of soil (**Figure 4**); meanwhile, the abundance of purple non-sulfur photosynthetic bacteria *Rhodobacter* increased significantly (**Figures 3A** **, C**). *Rhodobacter* can fix N_2_ and CO_2_ (based on the Calvin-Benson-Bassham cycle), although organic compounds are preferentially utilized over CO_2_ (Tabita, 1995; Tichi and Tabita, 2000). In addition, purple non-sulfur bacteria also have promoted functions of plant growth, such as phosphate dissolution (Sakarika et al., 2020), heavy metal repair (Batool et al., 2017; Sakpirom et al., 2017), and production of plant growth promoted compounds (Nunkaew et al., 2014).
(4) Enhancement of robustness in the bacterial co-occurrence network. In this study, the numbers of Gammaprotobacteria and Bacteroidetes as the hub nodes in the co-occurrence network of SS increased (**Figure 5**), and both these bacteria were considered to behave as symbiotrophic bacteria (Fierer et al., 2007; İnceoğlu et al., 2011; Semenov and Đukić, 2020). The network structure will be more stable and show more metabolic diversity, when these symbiotrophic bacteria as the core of network. In addition, the robustness analysis (**Figure 5**) also suggested that the treatment of SS makes the co-occurrence network structure more robust.

**Figure 7:**
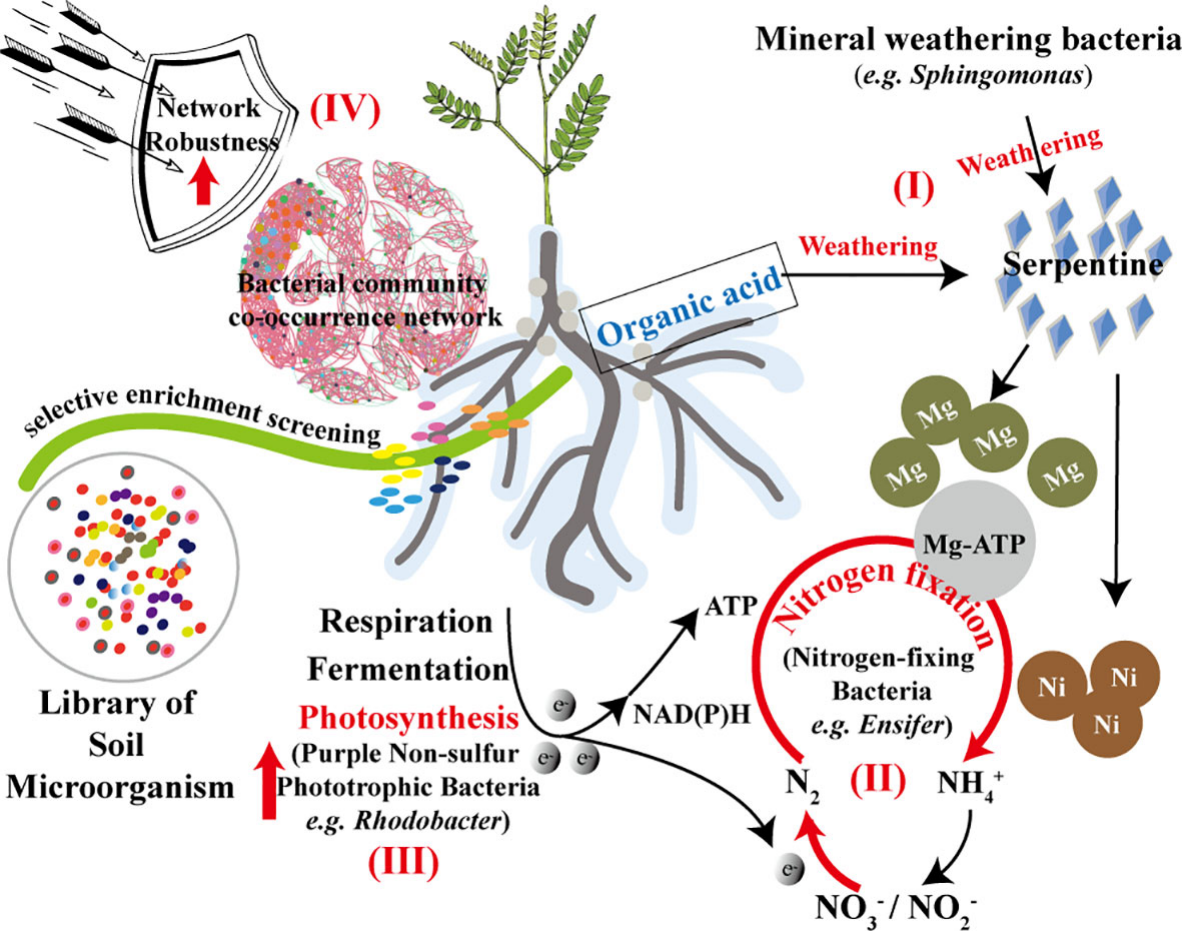
Functional prediction analysis of bacterial community selectively enriched in the rhizosphere of *S. cannabina* grows in CSS treated by application of organic fertilizer combined with river-sand and serpentine.

In summary, the positive development and functional analysis of the bacterial community in the rhizosphere of *S. cannabina* in SS are illustrated in **Figure 7**. The diagram highlights the soil bacterial action caused by the addition of serpentine, based on the application of organic fertilizer and river-sand.

Affected by the changes in soil physico-chemical properties, especially the weathering of serpentine (**Figure 7** **I**), many nitrate-reducing bacteria, nitrogen-fixing bacteria (**Figure 7** **II**), and phototrophic bacteria (**Figure 7** **III**) with promoted effects of plant growth were screened and enriched in the rhizosphere of *S. cannabina* in SS. Microorganisms can regulate bioavailable nitrogen by functions of nitrogen fixation and denitrification to exchange nitrogen with the atmosphere (Nelson et al., 2016), in which the enriched bacteria in SS can mobilize these two functions, which is conducive to improving the effect of nitrogen fixation in SS. In addition, the robustness of the co-occurrence network in SS was enhanced (**Figure 7** **IV**), which can better withstand the interference of external or internal uncertainties. Although no increase in inorganic carbon was detected in the sample of SS, but the positive development in rhizosphere bacterial community of *S. cannabina* was conducive to the improvement of CSS and growth of *S. cannabina*, which was of significance for improving the effect of carbon sink in soil.

## 5 Conclusion

In summary, the addition of organic fertilizer, river-sand and serpentine had a positive influence on the physical-chemical properties of coastal saline-alkali silt soil, the growth status of *S. cannabina*, and the construction of bacterial community in the rhizosphere of *S. cannabina*. A single application of organic fertilizer and the application of organic fertilizer combined with river-sand can improve CSS and promote the participation of rhizosphere bacterial community of *S. cannabina* in organic matter activation of soil. The application of organic fertilizer combined with river-sand and serpentine not only significantly embellished the saline-alkali status of soil, but also improved the proportion of soil base ions by supplementing the macro-element Mg and the trace element Ni; meanwhile, the beneficial bacterial flora with functions of promoting plant growth and N-cycle in the rhizosphere of *S. cannabina* could be enriched, while the robustness of the bacterial mutual co-occurrence network was increased; in addition, the synergistic effect of these additives could significantly promote the growth of *S. cannabina*, and increase soil organic matter content, so that CSS could be improved significantly. In general, the application of organic fertilizer and gravel combined with the cultivation of *S. cannabina* has significantly improved the coastal saline-alkali silt soil in this area. The research provides a theoretical basis and technical guidance for the development and utilization of CSS and development and growth of vegetation.

## Data availability statement

The datasets presented in this study can be found in online repositories. The names of the repository/repositories and accession number(s) can be found below: https://www.ncbi.nlm.nih.gov/, PRJNA869200.

## Author contributions

BL contributed to conceptualization, methodology, investigation, and supervision of the study, and contributed to writing - review & editing of the manuscript. XA wrote the original draft preparation and contributed to investigation, visualization, and data curation of the study. MS and KR contributed to investigation and visualization of the study. BL, MX and ZW contributed to project administration and funding acquisition of the study. YL and HL contributed to formal analysis and visualization of the study. All authors contributed to manuscript revision, read, and approved the submitted version.
