# Supplementary material for: Effect and mechanism of the improvement of coastal silt soil by application of organic fertilizer and gravel combined with Sesbania cannabina cultivation
Source: Front Plant Sci. 2022 Dec 22;13:1092089. doi: 10.3389/fpls.2022.1092089 (PMC9815860; doi:10.3389/fpls.2022.1092089)
Supplement: Supplementary file 1 [file DataSheet_1.docx]

**
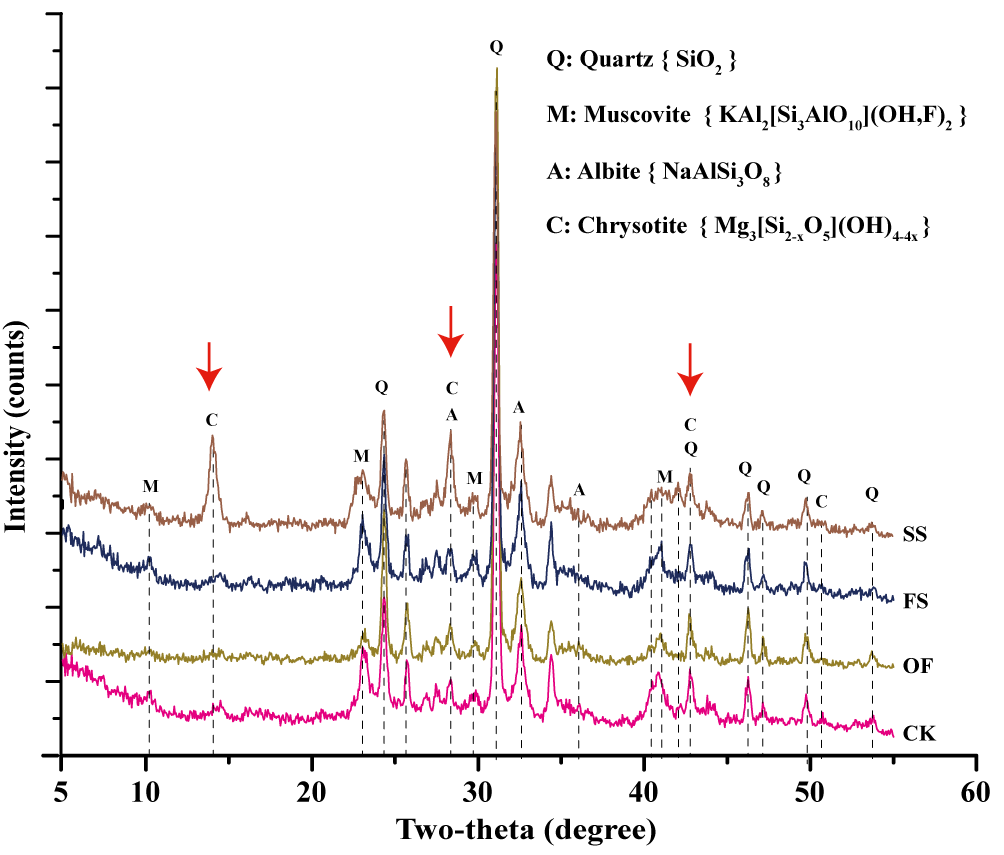
**

**Figure S1** Characterization of mineral crystal forms in soils with different treatments.

**
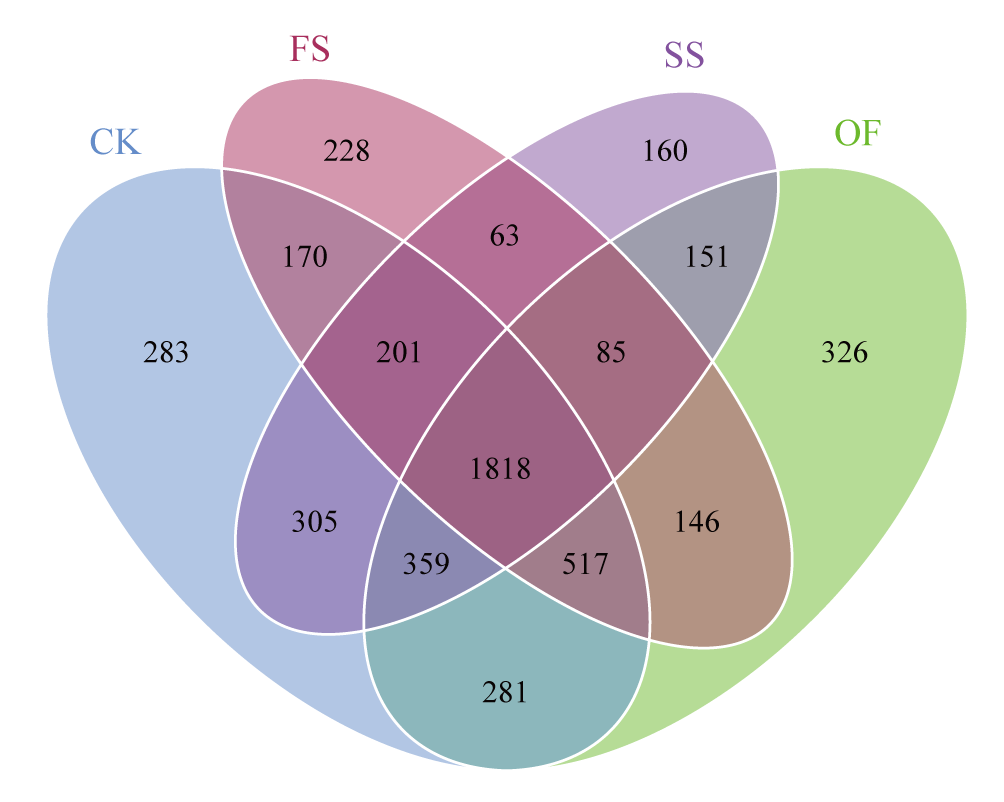
**

**Figure S2** Venn diagram shows the number of common and different OTUs among different samples

**Table S1** Predication function of dominant bacteria with significant difference among different samples

| Group1 *v.* Group2 | Taxa | Avg (Group1) | sd (Group1) | Avg (Group2) | sd (Group2) | *P*-value | level |
| --- | --- | --- | --- | --- | --- | --- | --- |
| CK *v.* OF | chemoheterotrophy | 21.3336% | 0.5413% | 24.6622% | 1.0054% | 0.0142 | ** |
|  | aerobic_chemoheterotrophy | 17.8465% | 0.6106% | 21.7448% | 1.7246% | 0.0468 | ** |
|  | cyanobacteria | 0.1509% | 0.0246% | 0.0994% | 0.0140% | 0.0476 | ** |
|  | oxygenic_photoautotrophy | 0.1509% | 0.0246% | 0.0994% | 0.0140% | 0.0476 | ** |
|  | photoautotrophy | 0.1509% | 0.0246% | 0.0997% | 0.0145% | 0.0480 | ** |
| OF *v.* FS | animal_parasites_or_symbionts | 1.5103% | 0.1570% | 3.6842% | 0.9088% | 0.0499 | ** |
|  | ureolysis | 1.0719% | 0.3280% | 2.7701% | 0.4108% | 0.0058 | *** |
|  | human_pathogens_all | 1.2711% | 0.1215% | 2.4408% | 0.5958% | 0.0712 | * |
|  | aromatic_compound_degradation | 0.8134% | 0.2526% | 1.6495% | 0.4159% | 0.0521 | * |
|  | predatory_or_exoparasitic | 0.1601% | 0.0068% | 0.1362% | 0.0018% | 0.0203 | ** |
| FS *v.* SS | nitrate_reduction | 2.7081% | 0.4844% | 5.3402% | 0.2353% | 0.0040 | ** |
|  | ureolysis | 2.7701% | 0.4108% | 2.0635% | 0.2795% | 0.0780 | * |
|  | phototrophy | 0.4595% | 0.0947% | 0.8083% | 0.2194% | 0.0941 | * |
|  | plastic_degradation | 0.4526% | 0.0557% | 0.1454% | 0.0714% | 0.0050 | *** |
|  | human_pathogens_pneumonia | 0.2219% | 0.0751% | 0.0235% | 0.0110% | 0.0420 | ** |
|  | dissimilatory_arsenate_reduction | 0.2187% | 0.0871% | 0.0635% | 0.0292% | 0.0785 | * |
|  | manganese_oxidation | 0.2162% | 0.0656% | 0.0222% | 0.0096% | 0.0337 | ** |
|  | human_pathogens_nosocomia | 0.2150% | 0.0660% | 0.0216% | 0.0107% | 0.0339 | ** |
|  | arsenate_detoxification | 0.2111% | 0.0872% | 0.0601% | 0.0275% | 0.0842 | * |
|  | hydrocarbon_degradation | 0.1525% | 0.0126% | 0.0781% | 0.0414% | 0.0789 | * |

**Note:** “*” denotes a P-value < 0.1, “**” denotes a P-value < 0.05, “***” denotes a P-value < 0.01.

**Table S2** Hub nodes of bacterial co-occurrence network of CK samples.

| Genus | Class | Degree | Betweeness centrality | Hub | Modularity_class |
| --- | --- | --- | --- | --- | --- |
| Catalinimonas | Bacteroidia | 30 | 1631.73 | 0.20 | A |
| Hahella | Gammaproteobacteria | 28 | 963.92 | 0.19 | A |
| Promicromonospora | Actinobacteria | 29 | 1592.95 | 0.19 | A |
| Sulfurimonas | Campylobacteria | 29 | 1592.95 | 0.19 | A |
| Desulfatiglans | Desulfobacteria | 30 | 1631.73 | 0.20 | A |
| Sumerlaea | Sumerlaeia | 29 | 1592.95 | 0.19 | A |
| JTB255_marine_benthic_group | Gammaproteobacteria | 29 | 943.12 | 0.19 | A |
| Domibacillus | Bacilli | 29 | 1297.65 | 0.19 | A |
| Gramella | Bacteroidia | 27 | 430.84 | 0.19 | B |
| Methylobacillus | Gammaproteobacteria | 27 | 299.91 | 0.18 | B |
| Prevotella | Bacteroidia | 29 | 1592.95 | 0.19 | B |
| Ruminococcus | Clostridia | 27 | 430.84 | 0.19 | B |
| Pelagibacterium | Alphaproteobacteria | 28 | 725.31 | 0.19 | B |
| Anaerovibrio | Negativicutes | 29 | 4774.06 | 0.13 | B |
| Arcobacter | Campylobacteria | 27 | 430.84 | 0.19 | B |
| X.Eubacterium._ruminantium_  group | Clostridia | 26 | 4.50 | 0.18 | B |
| Lachnospiraceae_UCG.003 | Clostridia | 30 | 1631.73 | 0.20 | B |
| Sutterella | Gammaproteobacteria | 30 | 1631.73 | 0.20 | B |
| Monoglobus | Clostridia | 30 | 1631.73 | 0.20 | B |
| Pseudorhodoplanes | Alphaproteobacteria | 29 | 1592.95 | 0.19 | B |
| Coprococcus | Clostridia | 29 | 1297.65 | 0.19 | B |
| Moraxella | Gammaproteobacteria | 29 | 943.12 | 0.19 | B |
| Succinivibrio | Gammaproteobacteria | 29 | 1592.95 | 0.19 | B |
| Planococcus | Bacilli | 26 | 3074.32 | 0.11 | B |
| UCG.004 | Bacilli | 26 | 3074.32 | 0.11 | B |

**Table S3** Hub nodes of bacterial co-occurrence network of OF samples.

| Genus | Class | Degree | Betweeness centrality | Hub | Modularity_class |
| --- | --- | --- | --- | --- | --- |
| Streptococcus | Bacilli | 26 | 2144.39 | 0.19 | A |
| Algoriphagus | Bacteroidia | 26 | 119.48 | 0.21 | A |
| Zeaxanthinibacter | Bacteroidia | 26 | 2821.02 | 0.20 | A |
| Agromyces | Actinobacteria | 27 | 184.51 | 0.21 | A |
| Lachnospiraceae_AC2044_  group | Clostridia | 26 | 126.02 | 0.20 | A |
| Actibacterium | Alphaproteobacteria | 27 | 206.39 | 0.21 | A |
| Haloferula | Verrucomicrobiae | 26 | 2144.39 | 0.19 | A |
| Isoptericola | Actinobacteria | 26 | 145.98 | 0.20 | A |
| Guyparkeria | Gammaproteobacteria | 26 | 2144.39 | 0.19 | A |
| Fulvivirga | Bacteroidia | 27 | 3846.11 | 0.19 | A |
| Limibaculum | Alphaproteobacteria | 26 | 3041.18 | 0.19 | A |
| Thioalkalispira.Sulfurivermis | Gammaproteobacteria | 26 | 119.48 | 0.21 | B |
| Hymenobacter | Bacteroidia | 25 | 311.35 | 0.18 | B |
| Aurantimonas | Alphaproteobacteria | 25 | 311.35 | 0.18 | B |
| Roseomonas | Alphaproteobacteria | 26 | 145.98 | 0.20 | B |
| Actibacter | Bacteroidia | 26 | 119.48 | 0.21 | B |
| Amphritea | Gammaproteobacteria | 26 | 145.98 | 0.20 | B |
| Halomonas | Gammaproteobacteria | 26 | 1312.34 | 0.17 | B |
| Cyclobacterium | Bacteroidia | 26 | 589.80 | 0.16 | B |
| Intrasporangium | Actinobacteria | 25 | 354.99 | 0.16 | B |
| Marinobacterium | Gammaproteobacteria | 26 | 1312.34 | 0.17 | B |

**Table S4** Hub nodes of bacterial co-occurrence network of FS samples.

| Genus | Class | Degree | Betweeness centrality | Hub | Modularity_class |
| --- | --- | --- | --- | --- | --- |
| Anaerococcus | Clostridia | 31 | 4026.99 | 0.16 | A |
| Gallicola | Clostridia | 27 | 61.33 | 0.17 | A |
| Peptoniphilus | Clostridia | 31 | 2023.68 | 0.19 | A |
| Clostridium_sensu_stricto_1 | Clostridia | 29 | 1869.08 | 0.16 | A |
| Peptostreptococcus | Clostridia | 33 | 1620.09 | 0.18 | A |
| Romboutsia | Clostridia | 31 | 887.60 | 0.18 | A |
| Staphylococcus | Bacilli | 32 | 727.25 | 0.18 | A |
| Turicibacter | Bacilli | 28 | 821.16 | 0.14 | A |
| NK4A214_group | Clostridia | 28 | 821.16 | 0.14 | A |
| UCG.002 | Clostridia | 31 | 1456.94 | 0.19 | A |
| Terrisporobacter | Clostridia | 28 | 511.70 | 0.17 | A |
| Actinomyces | Actinobacteria | 30 | 1072.38 | 0.18 | A |
| Anaerovibrio | Negativicutes | 28 | 779.93 | 0.16 | A |
| Family_XIII_AD3011_group | Clostridia | 28 | 589.62 | 0.17 | A |
| Solobacterium | Bacilli | 28 | 821.16 | 0.14 | A |
| Blautia | Clostridia | 25 | 128.75 | 0.11 | A |
| Kurthia | Bacilli | 27 | 61.33 | 0.17 | A |
| Sulfurimonas | Campylobacteria | 31 | 1456.94 | 0.19 | A |
| Macrococcus | Bacilli | 31 | 1456.94 | 0.19 | A |
| Murdochiella | Clostridia | 30 | 222.22 | 0.18 | A |
| Erysipelatoclostridium | Bacilli | 28 | 511.70 | 0.17 | A |
| Coprococcus | Clostridia | 25 | 2268.15 | 0.10 | A |
| Exiguobacterium | Bacilli | 27 | 63.89 | 0.14 | A |
| Corynebacterium | Actinobacteria | 28 | 100.61 | 0.15 | A |
| Trueperella | Actinobacteria | 28 | 100.61 | 0.15 | A |
| Ezakiella | Clostridia | 26 | 1395.40 | 0.12 | A |
| Aeromonas | Gammaproteobacteria | 26 | 1395.40 | 0.12 | A |
| Helcococcus | Clostridia | 26 | 1395.40 | 0.12 | A |
| Pseudomonas | Gammaproteobacteria | 28 | 589.62 | 0.17 | B |
| Cellvibrio | Gammaproteobacteria | 29 | 168.10 | 0.18 | B |
| Cyclobacterium | Bacteroidia | 31 | 1456.94 | 0.19 | B |
| unidentified_Saccharimonadales | Saccharimonadia | 28 | 589.62 | 0.17 | B |
| Anderseniella | Alphaproteobacteria | 27 | 286.33 | 0.13 | B |
| Actibacterium | Alphaproteobacteria | 27 | 2351.28 | 0.13 | B |
| unidentified_Sandaracinaceae | Polyangia | 28 | 3367.04 | 0.14 | B |
| Kineococcus | Actinobacteria | 28 | 100.61 | 0.15 | B |

**Table S5** Hub nodes of bacterial co-occurrence network of SS samples.

| Genus | Class | Degree | Betweeness centrality | Hub | Modularity_class |
| --- | --- | --- | --- | --- | --- |
| unidentified_Chloroplast | Cyanobacteriia | 25 | 439.49 | 0.20 | A |
| Kocuria | Actinobacteria | 25 | 439.49 | 0.20 | A |
| Sumerlaea | Sumerlaeia | 25 | 290.14 | 0.16 | A |
| JTB255_marine_benthic_group | Gammaproteobacteria | 25 | 290.14 | 0.16 | A |
| Tumebacillus | Bacilli | 25 | 290.14 | 0.16 | A |
| Pseudohongiella | Gammaproteobacteria | 24 | 383.26 | 0.18 | A |
| Gallicola | Clostridia | 24 | 707.74 | 0.14 | B |
| UCG.005 | Clostridia | 24 | 72.25 | 0.10 | B |
| Clostridium_sensu_stricto_1 | Clostridia | 24 | 1831.70 | 0.18 | B |
| Romboutsia | Clostridia | 25 | 439.49 | 0.20 | B |
| Roseburia | Clostridia | 24 | 1782.81 | 0.16 | B |
| Lachnospira | Clostridia | 25 | 268.32 | 0.18 | B |
| X.Eubacterium._siraeum_group | Clostridia | 24 | 3185.14 | 0.17 | B |
| Sutterella | Gammaproteobacteria | 25 | 665.53 | 0.16 | B |
| Niabella | Bacteroidia | 24 | 72.25 | 0.10 | B |
| Chitinophaga | Bacteroidia | 24 | 1782.81 | 0.16 | B |
| Coprococcus | Clostridia | 25 | 268.32 | 0.18 | B |
| Oligoflexus | Oligoflexia | 24 | 341.56 | 0.11 | B |
| Quadrisphaera | Actinobacteria | 25 | 439.49 | 0.20 | B |
| Bacillus | Bacilli | 24 | 341.56 | 0.11 | B |
| Marmoricola | Actinobacteria | 25 | 950.73 | 0.17 | B |
| X.Eubacterium._xylanophilum_  group | Clostridia | 24 | 7367.65 | 0.12 | B |
| Anaerovibrio | Negativicutes | 25 | 290.14 | 0.16 | B |
| Moraxella | Gammaproteobacteria | 25 | 290.14 | 0.16 | B |
| W5053 | Clostridia | 25 | 290.14 | 0.16 | B |
| Anderseniella | Alphaproteobacteria | 25 | 290.14 | 0.16 | B |
| Methylophilus | Gammaproteobacteria | 24 | 1040.97 | 0.19 | B |
| Caulobacter | Alphaproteobacteria | 24 | 3428.63 | 0.20 | B |
| Ellin6067 | Gammaproteobacteria | 25 | 280.88 | 0.20 | B |
| Lachnospiraceae_AC2044_  group | Clostridia | 24 | 1040.97 | 0.19 | B |
| Monoglobus | Clostridia | 24 | 383.26 | 0.18 | B |
| Faecalibacterium | Clostridia | 24 | 1040.97 | 0.19 | B |
| Thioalkalispira.Sulfurivermis | Gammaproteobacteria | 24 | 72.25 | 0.10 | C |
| Planococcus | Bacilli | 24 | 72.25 | 0.10 | C |
| Persicitalea | Bacteroidia | 24 | 72.25 | 0.10 | C |
